# Trajectory Analysis of Suicidal Ideation in Spanish College Students Using Ecological Momentary Assessment

**DOI:** 10.3389/fpsyt.2022.853464

**Published:** 2022-03-31

**Authors:** José Enrique Layrón Folgado, Andrea Conchado Peiró, José H. Marco, María Luisa Barrigón, Enrique Baca-García, Sandra Pérez Rodríguez

**Affiliations:** ^1^Escuela de Doctorado, Universidad Católica de Valencia San Vicente Mártir, Valencia, Spain; ^2^Department of Statistics and Applied Operations Research and Quality, Polytechnic University of Valencia, Valencia, Spain; ^3^Department of Personality, Evaluation and Psychological Treatment, University of Valencia, Valencia, Spain; ^4^CIBER Fisiopatología Obesidad y Nutrición (CIBEROBN), Madrid, Spain; ^5^Department of Psychiatry, Jiménez Díaz Foundation Hospital, Madrid, Spain; ^6^Department of Psychiatry, University Hospital Virgen del Rocio, Seville, Spain; ^7^Department of Psychiatry, University Hospital Rey Juan Carlos, Mostoles, Spain; ^8^Department of Psychiatry, General Hospital of Villalba, Madrid, Spain; ^9^Department of Psychiatry, University Hospital Infanta Elena, Valdemoro, Spain; ^10^Department of Psychiatry, Madrid Autonomous University, Madrid, Spain; ^11^CIBERSAM (Centro de Investigacion en Salud Mental), Carlos III Institute of Health, Madrid, Spain; ^12^Departamento de Psicología, Universidad Catolica del Maule, Talca, Chile; ^13^Department of Psychiatry, Centre Hospitalier Universitaire de Nîmes, Nîmes, France

**Keywords:** suicidal ideation, suicide, college students, ecological momentary assessment (EMA), interpersonal theory for suicide

## Abstract

**Introduction:**

Suicide is a preventable death in young people. It is well known that suicide behavior is a multicausal phenomenon. However, suicidal ideation (SI) commonly underlies suicide, and Ecological Momentary Assessment (EMA) can help us to better characterize it and its risk and protective factors in the short term. We aimed, first, to investigate the estimated prevalence and trajectories of SI in a community sample of Spanish college students using an EMA methodology and, second, explore the associations between risk and protective factors and SI categorized as moderate or low.

**Materials and Methods:**

A total of 737 participants followed the EMA during a period of 6 months. We estimated the prevalence and trajectories of SI and the associations between depressive symptoms, positive and negative affect, thwarted belongingness, perceived burdensomeness, cognitive reappraisal, emotional suppression, and purpose in life with the MEmind smartphone App. SI was assessed 14 times during this period.

**Results:**

Twenty-eight participants referred to SI at least once in longitudinal assessments. We found a lack of curvature and, thus, a relatively stable trajectory of SI. Two groups of latent dimensions were observed related to risk and protective factors of SI. One latent dimension of the risk factors (higher levels of thwarted belongingness, perceived burdensomeness, depressive symptoms, negative affect, and emotional suppression) best represented the group with moderate levels of SI, and a second latent dimension of protective variables (positive affect, cognitive reappraisal, and purpose in life) best represented the group with lower levels of SI.

**Discussion:**

These findings may indicate that students with a sense of having a life worth living, in addition to having the ability to reevaluate their negative beliefs, are less likely to experience high levels of SI. Therefore, purpose in life would be a protective factor against the presence of SI.

## Introduction

According to the World Health Organization (WHO), around 700,000 people die annually as a result of suicide, and it is the fourth leading cause of death among young people between 15 and 19 years old (1). Therefore, it is a worrisome behavior that has increased in recent years (2). Although suicidal behavior is a multi-factorial phenomenon (3), and a great deal of knowledge about suicide risk and protective factors has been obtained in recent years (4), less is known about the direct precursors of suicidal behavior (5).

Most of the clinical correlates of suicide seem to be best conceptualized as correlates of suicidal ideation (SI) (6). Research has found that SI is one of the strongest predictors of suicide attempts (SA) (7), and even more so in the presence of previous suicide attempts and non-suicidal self-injury (NSSI) (8). Moreover, it is important to point out that the occurrence of these kinds of ideas can be chronic (9).

Furthermore, SI increases exponentially during adolescence and remains high from then on (10). In this regard, in the United States, in 2017, about 17% of adolescents seriously considered committing suicide (11). A recent meta-analysis revealed a high persistence of adolescent-onset suicidal thoughts and behaviors into the college years (12). At the same time, rates of lifetime SI are higher in females than in males (13), and completed suicide is more prevalent in men (14). Across all countries, 60% of the evolution from SI to planning or SA takes place within the first year after SI onset (15).

Recent literature has found neurocognitive functions that are associated with the risk of suicide behavior in young people (16, 17). In this regard, SI usually starts in adolescence and is associated with both clinical and community populations (18), with its intensity fluctuating considerably over short periods of time (19). Furthermore, it is relevant to explore this variation because an examination over long periods (months or years) cannot capture the true nature of these thoughts. A recent study that focused on the longitudinal evolution of SI using Ecological Momentary Assessment (EMA) showed that almost a third of all SI scores were one standard deviation below or above the previous response only 4–8 h earlier (20). Moreover, a recent theoretical study (21) proposed that there may be a minimum of two subtypes of the experience of SI. One suggested subtype is described as large fluctuations in SI severity in reaction to life stress (stress responsive ideators). The other suggested subtype is described as persistent levels of SI that do not fluctuate in reaction to life events (non-stress responsive ideators). In addition, Kleiman et al. (22) found five phenotypes of SI that differed mainly in the average severity of SI over time (Low mean, low variability; Moderate mean, low variability; Moderate mean, moderate variability; High mean, moderate variability; and High mean, high variability). Moreover, there is a promising strategy for exploring suicide that focuses on the evaluation of “*serious suicide attempt survivors*.” These would include cases where people received quick medical attention through speedy and effective first-aid care, cases that received other adequate forms of emergency treatment, or even cases where the response was due to mere coincidence (23). Therefore, this point of view emphasizes the differences between levels of suicidal behaviors in people at high risk for suicide.

The Interpersonal Theory of Suicide (24) proposes two interpersonal variables that may lead a person to SI: thwarted belongingness and perceived burdensomeness. The former points out the person’s need to be socially integrated, whereas the latter describes the person’s perception of being a burden to others. The presence of only one of these variables leads to passive SI, whereas the combined presentation of both along with hopelessness may lead to active SI (25). Recent research found a significant direct effect between thwarted belongingness, perceived burdensomeness, and hopelessness on college students with a presence of SI (26). Otherwise, SI is related to affective symptomatology in general and in young people in particular (27). Recent research revealed that high negative affect and low positive affect show a significant association with SI in adolescents (28). Furthermore, negative emotional states affect SI more strongly than factors such as low social integration (29).

In addition, emotional dysregulation seems to be related to an elevated suicidal desire and may participate in the conversion to suicidal behavior in people who use dysfunctional coping methods (30). Expressive suppression is a strategy included in the model of emotion regulation postulated by Gross and John (31). Importantly, studies have found that increased levels of expressive suppression may significantly predict SI (32). In the opposite direction, other studies have shown that people who modulate their emotions using a cognitive reappraisal strategy show lower levels of SI and suicidal behaviors than those who have trouble using this strategy (33). In this regard, different studies have shown that high levels of cognitive reappraisal are related to less suicidality (32, 34).

Regarding protective variables against SI, meaning in life has been found to be a protective and mediating factor that can reduce SI (35, 36). Meaning in life refers to the fundamental motivational force of the human being and the condition of personal self-realization (37), and it has been described as comprising three main dimensions: purpose, coherence, and significance (38). Moreover, some studies have identified a link between low meaning in life and suicide risk factors (39, 40). In addition, positive affect has been shown to be a significant barrier against SI, given that this variable is negatively related to SI (28).

It should be noted that, in contrast with the moderately static outcome incidence, SI trajectories are a dynamic result and, therefore, need a more fine-grained assessment (41). Previous literature has demonstrated that fluctuation is frequent and clinically significant (42, 9). In this regard, the recurrence of SI may have an important impact on other clinical outcomes, and so it is important to consider SI trajectories over time. Czyz and King (42) showed that a group of chronic SI adolescents reported higher levels of hopelessness, even after controlling other variables such as depressive symptoms. Furthermore, Wolff et al. (41) found that emotion dysregulation considerably differentiated people with chronic SI from people with declining SI because those who were more unconcerned about emotional responses and had inadequate contact with emotion regulation strategies were more likely to report chronic SI. Moreover, a four-year longitudinal study showed that thwarted belongingness amplified the projection of SI trajectories in the outcome of suicide loss, and perceived burdensomeness predicted complicated-grief levels over time in suicide survivors (43).

### Ecological Momentary Assessment and Suicidal Ideation

Studies based on EMA (44) might be especially relevant for evaluating SI at the moment it occurs (45). Moreover, the time variable is particularly important in the study of suicidal behavior, given that numerous studies have shown that the first 6 months is the critical period for carrying out a new attempt (46). Therefore, examination of SI over long periods of time (months or years) cannot capture the true nature of these thoughts (20).

Furthermore, there is evidence of increasing research on EMA and suicidality [e.g., (15, 19, 20, 47–50)]. Although these studies have made noteworthy contributions, there are still important gaps related to the use of EMA with SI and SA. In this regard, the majority of EMA studies have included a fairly short follow-up period (one or two weeks), primarily with adults (45, 51).

Although EMA studies in college samples are relatively extensive, with contributions in areas such as physical activity (52), food choices (53), sleep quality (54), marijuana (55), alcohol (56), or the effects of mindfulness training on daily stress (57), there is a lack of studies that specifically evaluate SI and SA in college students using this methodology.

Accordingly, the aim of this study was to: (1) investigate the estimated prevalence and trajectories of SI in a community sample of Spanish college students using EMA methodology for a period of 6 months and (2) explore the associations between risk and protective factors of SI—depressive symptoms, positive and negative affect, cognitive reappraisal, emotional suppression, thwarted belongingness, perceived burdensomeness, and purpose in life- and their relationships with categories of high, moderate, or low SI in a community sample of university students.

Regarding the first objective, we expected to find an estimated prevalence of around 10%, based on previous literature (58). Furthermore, we expected to find a high fluctuation in SI over short periods of time, in accordance with results from Hallensleben et al. (48). As for the second objective, we hypothesized that risk factors would better represent the group of participants with high or moderate levels of SI, and a second latent dimension of protective variables would better represent the group of patients with lower levels of SI.

## Materials and Methods

### Participants

In a context of voluntary participation, 975 undergraduates were initially approached, of whom 737 completed the initial evaluation (75.59%). They were recruited from a Spanish university and received instruction about how to use the EMA in their Smartphones with the “MEmind” app (59).

Participants used the EMA for a period of six months; 54.95% were women (*n* = 405), and 45.05% were men (*n* = 332). The majority were young (mean = 23 years, SD = 5.7), although there was a wide age range (17–65 years). In addition, 55.1% (*n* = 406) of the participants were single; 41.2% (*n* = 304) had a partner, and 3.7% (*n* = 27) were separated.

The EMA protocol was composed of a series of evaluations in different periods of time. In this regard, 737 participants completed the initial evaluation, whereas in the third evaluation (*n* = 509), students completed the assessment, of whom 58% were women (*n* = 295) and 42% men (*n* = 214). Moreover, during the sixth evaluation, (*n* = 275) participants completed the assessment, of whom 61% were women (*n* = 168), and 39% were men (*n* = 107). In the ninth evaluation, we had responses from a total of *n* = 132 students, of whom 70% were women (*n* = 93), and 30% were men (*n* = 39), whereas in the 12th evaluation (*n* = 44), participants completed the assessment, of whom 86% were women (*n* = 38), and 14% were men (*n* = 6).

The inclusion criteria were being a college student, having and using a Smartphone, and understanding and agreeing with the working procedure. The exclusion criterion was refusing to participate. The procedure in the present study was approved by the ethical committee of the Catholic University of Valencia (code 072). The participants were recruited in their classrooms and signed informed consents before the initial evaluation. They received a certificate of participation and university credits as compensation. The students were recruited between September 2019 and January 2020. For privacy reasons, no personal information was collected that could associate the ID of the Smartphone with the identity of the participant in this study. The sample of college students completed a subset of self-report questionnaires, described below. The database was registered and protected with the highest level of security, in accordance with the Spanish data protection law.

### Assessments and Measures

*Ecological Momentary Assessment* (EMA) *SI questions*. This tool was designed *ad hoc* to assess suicide risk with a dynamic non-linear model that combines Likert-type scales and visual analog scales. The questions about suicide behavior were the following: (1) During the past week, have you self-injured with the intention of hurting yourself (I have made cuts, blows, stuck needles, burned myself, hit myself, scratched my skin forcefully, not allowed wounds to heal, I have ingested substances, etc.)? (yes–no). If the answer is yes, how often did you do so? (2) In the past week, have you thought about taking your life? If the answer is yes, how often did you do so? (3) In the past week, have you planned to kill yourself? (yes-no), If the answer is yes, how often did you do so? (4) In the past week, have you tried to kill yourself? (yes–no). If the answer is yes, how often did you do so?

*The Emotion Regulation Questionnaire* (ERQ) (60). This scale is the Spanish version of the ERQ (31). It consists of 10 items divided into two subscales: cognitive reappraisal (6 items) and emotional suppression (4 items), and participants respond on a 7-point Likert-scale. The ERQ showed reliability for the reappraisal scale (α = 0.75) and for suppression (α = 0.79). In our sample, there was adequate internal consistency for cognitive reappraisal (ϖ = 0.78) and emotional suppression (ϖ = 0.78).

*The Positive and Negative Affect Schedule* (PANAS) (61). This scale is the Spanish version of the PANAS (62), and it consists of 20 positive and negative adjectives rated on a 5-point Likert scale. PANAS showed positive reliability for positive affect (α = 0.76) and negative affect (α = 0.83). In our sample, there was adequate internal consistency for positive affect (ϖ = 0.86) and negative affect (ϖ = 0.85).

*Interpersonal Needs Questionnaire* (INQ) (63). This scale is the Spanish version of the INQ (64), and it consists of 15 items divided into two subscales: (1) thwarted belongingness and (2) perceived burdensomeness. Spanish scale reliabilities were high for perceived burdensomeness (α = 0.96) and thwarted belongingness (α = 0.78). In our sample, there was adequate internal consistency for thwarted belongingness (ϖ = 0.81) and perceived burdensomeness (ϖ = 0.83).

*Patient Health Questionnaire* (PHQ-9) (65). This scale is the Spanish version of the PHQ-9 (66), and it consists of nine items with a total score between 0 and 27 that indicates the presence of depressive symptoms in the past two weeks. The original version has a sensitivity of 75% and a specificity of 90%. The Spanish version has an overall accuracy of 88%; sensitivity, 87%; and specificity, 88%, similar to the original questionnaire. In our sample, there was adequate internal consistency (ϖ = 0.72).

*Purpose In Life-10* (PIL-10) (67). This scale is a reduced Spanish version of the PIL (68), and it is composed of a 10-item Likert scale related to different aspects of meaning in life. The total score ranges from 10 to 70, so that higher scores show greater meaning in life. The PIL-10 showed high reliability for the global scale (α = 0.92) (67). In our sample, there was adequate internal consistency for the PIL-10 (ϖ = 0.88).

### Procedure

To verify the feasibility of the methodology and its correct enactment, a pilot study was designed for subsequent implementation on a larger scaler. This study was carried out with 60 students who answered the tests that made up the final evaluation protocol. The pilot study lasted approximately 30–35 min. With the pilot study, any doubts about the language used in the questionnaires were clarified, as well as questions about use and access to the MEmind application.

First, during the initial evaluation, the information corresponding to each participant was collected based on the responses to the questionnaires related to depressive symptoms, positive and negative affect, cognitive reappraisal, emotional suppression, thwarted belongingness, perceived burdensomeness, and purpose in life. Second, the MEmind application sent a notification to the participants’ mobile devices asking them to participate daily by answering the different SI questions, with a total of 14 different measurements over a 6-month period.

For sample recruitment, the research team first contacted the deans by email. In most of the cases, the dean sent the evaluation request to the different professors of each course in the different majors, and each professor proposed a suitable time for the initial evaluation. During class time, the researchers explained the objective of the investigation, and if the students agreed, they were instructed to sign the informed consent. The initial evaluation took an average of 20 min to complete, and any questions about the procedure were answered by the researchers. The researchers encouraged participants to sit apart from their classmates in order to avoid sharing the content of their answers through their Smartphones and facilitate honesty on sensitive items. Similarly, researchers helped participants on a case-by-case basis when there were difficulties in accessing the application, mainly due to not updating their Smartphone devices or to problems involving Internet access, which is required to download the application.

### Assessment With the “MEmind” App

MEmind is a web application created to fuse different data sources. It is accessible at www.memind.net, and it is compatible with Tablets, computers, and Smartphones with any operating system. The application creates a database that is accessed with a personalized and unidentified password, so that the participants can record their answers to the diverse blocks of questions personally in their own environment. Data and keys are hidden by a key management infrastructure that executes strict logical and physical security controls to avoid unauthorized entrance (59, 69).

First, initial data were obtained in the participants’ university through the questionnaires that made up the study assessment protocol. Second, after the first assessment, they were asked to participate daily by answering the different questions presented by MEmind through a notification on the participants’ mobile devices. The application’s questions consisted of the items on the different questionnaires, presented at different times of the day and during the week. The protocol also provided a section for notes where participants could report qualitative information about their personal situation and ask for individualized psychological help. Moreover, the application was presented through the Smartphones based on two types of questions: static questions that appeared every day at the same time or dynamic questions that changed randomly over time.

The number of questions was progressively reduced throughout the follow-up to ensure feasibility and reduce dropouts. During the first month, the participants had to answer four blocks of questions per day, two static items and two items from a dynamic random selection of the overall questions. To guarantee a balanced evaluation of the variables, all the items were used before starting a new round of questions. During the second and third month, the students were evaluated through simple questions twice a day. Finally, during the last three months of follow-up, participants were only evaluated four times a week with dynamic items that they had to respond to in the next half hour. Given that this paper is part of a larger study with an EMA protocol with broader objectives, for this study we selected the EMA questions related to SI, in order to explore their longitudinal trajectories. In this regard, participants did not receive any indications about emotion management during the EMA. However, in cases where the participants anonymously reported the presence of suicidal behavior, a contact number was provided so that the person could receive psychological treatment. However, none of the participants asked for the help offered.

### Data Analysis

**FIGURE 1:**
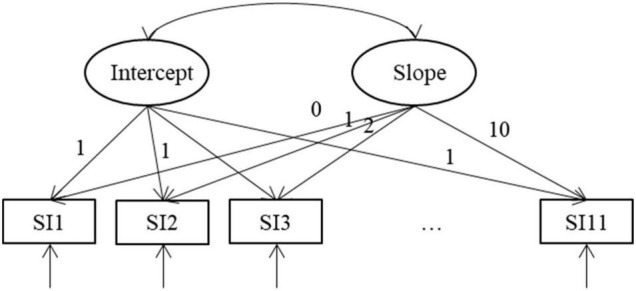
Growth mixture model (GMM) for suicidal ideation (SI).

A growth mixture model (GMM) was specified to gain a better understanding of the underlying populations according to their SI trajectories. As Figure 1 shows, the unconditional first-order GMM model hypothesized that two latent factors (intercept and slope) had a direct effect on 11 equally spaced points of measurement of frequency of SI.

This model assumes that the distribution of the repeated measures of SI frequency is a finite mixture of p—dimensional normal probability distribution, as follows:


$$f\,\left(y_{i}\right)=\sum_{k=1}^{K}\pi_{k}\,\phi_{k}\,\left(\Lambda_{k}\,\alpha_{k},\Lambda_{k}\,\Psi_{k}\,\Lambda_{k}^{\prime }+\Theta_{k}\right)$$

Where y_i_ is the vector containing 11 repeated measures of frequency of SI per participant, π_k_ represents the probability that a participant belongs to class k, Λ_k_ is a matrix of factor loadings (intercept and slope), α_k_ is a vector holding the means of individual trajectory parameters (between—class component), Ψ_k_ is a residual matrix reflecting individual differences (ζ_i_) from these means (within—class component), and Θ_k_ is a covariance matrix for measurement errors (ε_i_).

In order to examine the goodness of fit of this model, we compared an unconditional univariate single-class growth model with other models with two and three classes, using the entropy statistic, as well as the following indexes: the Akaike Information Criterion (AIC), the Bayesian Information Criterion (BIC), the Sample-Size Adjusted BIC (SSBIC), the nested model likelihood ratio suggested by Lo, Mendell, and Rubin (LMR), the Vuong, Lo, Mendell, and Rubin (VLMR), and the bootstrapped likelihood ratio test (BLRT). We also considered the parsimony criteria, the meaning and interpretability of the solution, and the recommendations provided by Nylund et al. (70) concerning the sensitivity of these indexes to small sample sizes. Once individuals were classified according to their trajectories, we examined the association between these classes and participants’ health and psychological strategies. We performed an exploratory factor analysis using maximum likelihood estimation and varimax rotation, and we summarized the results through a biplot graphical representation.

## Results

*N* = 737 (75.59%) students completed the subset of questionnaires at initial evaluation through the MEmind app, and 28 participants (3.8%) reported SI at least once during the EMA follow-up (14 moments of measurement). To assess trajectories across time, we estimated the average number of ideations through the middle point of the class interval for each participant and measurement (None = 0, Once = 1, From 2 to 4 = 3, More than 5 = 5) (Table 1).

**TABLE 1:** Relative frequencies and attrition rate.

	1	2	3	4	5	6	7	8	9	10	11	12	13	14
None (%)	57	82	52	68	61	69	71	82	75	75	67	100	50	100
Once (%)	21	14	40	21	28	25	21	9	13	0	17	0	0	0
From 2 to 4 (%)	18	4	8	5	6	6	0	0	13	0	0	0	0	0
More than 5 (%)	4	0	0	5	6	0	7	9	0	25	17	0	50	0
Attrition rate (%)	0	0	11	32	36	43	50	61	71	71	79	89	93	96

*How many times have you thought about ending your life/suicide (since the last measurement)?*

To examine the nature of the mean-level change, the analysis of a GMM was carried out for the frequency of SI, as Table 2 shows. Although we could not confirm a significant improvement in goodness-of fit indexes due to the small sample size, the number of classes provided successive improvements in the AIC, BIC, SSBIC indices and entropy. The model with three classes was eventually selected because it presented the best fit and the solution was meaningful in terms of the estimated parameters. The four-class model could not reach convergence during the estimation process.

**TABLE 2:** Fit indices for one- to three-class growth mixture models (unconditional).

	One class	Two classes	Three classes
H0 Value	–183.0	–171.9	–155.7
H0 Scaling Correction Factor for MLR	1.61	1.46	1.3545
AIC	398.0	381.7	353.4
BIC	419.4	407.1	381.4
SSBIC	369.6	348.0	316.1
Entropy	–	0.841	0.913
VLMR *p* value	–	0.3786	0.1978
LMR *p* value	–	0.3932	0.2135
BLRT *p* value	–	0.000	0.1034
Proportions for the latent classes	100%	7.2% (*n* = 2)	3.6% (*n* = 1)
		92.8% (*n* = 26)	67.8% (*n* = 19)
			28.6% (*n* = 8)

*AIC, Akaike Information Criterion; BIC, Bayesian Information Criterion; SSBIC, sample size adjusted Bayesian Information Criterion; VLMR, Vuong–Lo–Mendell–Rubin test; LRT, Lo–Mendell–Rubin test; BLRT, Bootstrapped–Lo–Mendell–Rubin test.*

Table 3 shows growth parameter estimates for the final unconditional model composed of three classes. Two of the classes (two and three) presented plain trajectories with non-significant slope parameters, whereas the first class was composed of only one participant who was considered an extreme value. The majority of the participants were assigned to a class with relatively low frequencies of SI.

**TABLE 3:** Growth factor parameter estimates for three-class conditional model.

Class number		Intercept mean (SE)	Slope mean (SE)
1	Extreme value (3.6%)	1.064 (0.109)*	0.270 (0.015)*
2	Low (67.8%)	0.214 (0.038)*	–0.015 (0.008)
3	Moderate (28.6%)	0.822 (0.065)*	–0.052 (0.035)

*p < 0.001 (two-tailed).

**FIGURE 2:**
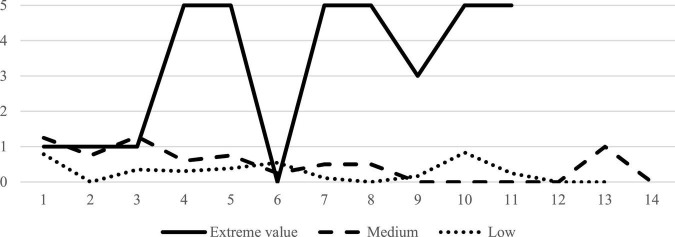
Average number of suicidal ideations per class across time (14 measurements).

The second largest class was characterized by participants with moderate and stable levels of SI frequency, as depicted in Figure 2. Next, principal component analysis was applied to the following measures: Cognitive reappraisal (ERQ), emotional suppression (ERQ), purpose in life (PIL), thwarted belongingness (INQ), perceived burdensomeness (INQ), positive affect (PANAS), negative affect (PANAS), and depressive symptoms (PHQ-9).

Based on the Kayser criterion retention, two latent factors accounted for 68% of the total sample variance. As Table 4 reveals, the first factor was labeled ‘Risk factors’ because it showed high factor loadings (greater than 0.5) on the Patient Health Questionnaire (PHQ-9), the Expressive Suppression subscale of the Emotion Regulation Questionnaire (ERQ), both the Burden and Belonging subscales of the Interpersonal Needs Questionnaire (INQ), and the Negative Affect Schedule (PANAS). The second factor represents a collection of positive factors and was labeled “Protective factors,” with the following total scores showing factor loadings greater than 0.5: Cognitive reevaluation from the Emotion Regulation Questionnaire (ERQ), the Purpose in Life (PIL) scale, and the Positive Affect Schedule (PANAS) (Table 4).

**TABLE 4:** Factor loadings of participants’ health and psychological strategies.

	Factor	
	1	2
Depressive Symptoms (PHQ-9)	**0.807**	–0.264
Cognitive Reappraisal (ERQ)	–0.235	**0.681**
Emotional Suppression (ERQ)	**0.624**	–0.14
Purpose in Life (PIL-10)	–0.375	**0.781**
Perceived Burdensomeness (INQ)	**0.769**	–0.387
Thwarted Belongingness (INQ)	**0.579**	–0.506
Positive Affect (PANAS)	0.048	**0.914**
Negative Affect (PANAS)	**0.916**	0.042

*first factor (risk factors), second factor (protective factors). Bold values refer to total scores showing factor loadings greater than 0.5.*

**FIGURE 3:**
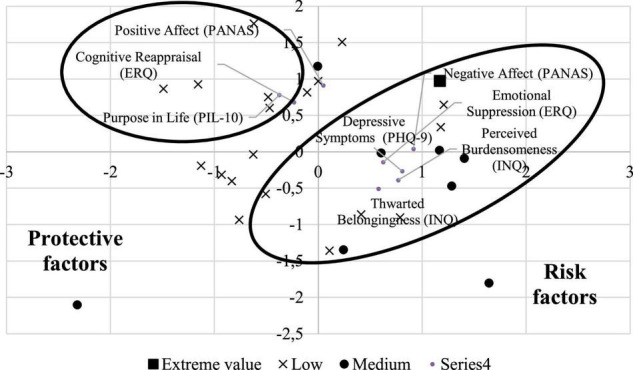
Biplot of factor scores and loadings obtained for participants’ health and strategies and their classification according to the frequency of suicidal ideation.

The graphical representation of the factor loadings and scores in Figure 2 allowed us to identify some interesting relationships between participants’ health and strategies and their classifications according to their trajectories across time with regard to SI. As Figure 3 shows, the collection of strategies whose high loadings in the “Risk factors” dimension was close to individuals with moderate levels of SI’. Similarly, the location of “Protective factors” in the graph was close to the majority of participants with low levels of frequency in SI. The extreme value identified as an independent class was not clearly located close to any of these dimensions.

## Discussion

This study had two main objectives. The first was to investigate the estimated prevalence and trajectories of SI in a community sample of Spanish college students using EMA methodology. The second was to explore the associations between risk and protective factors—depressive symptoms, positive and negative affect, cognitive reappraisal, emotional suppression, thwarted belongingness, perceived burdensomeness, and purpose in life- and their relationships with categories of high, moderate, or low SI in a community sample of university students.

In relation to the first objective, the percentage of college students who presented SI in our study was 3.80%. This figure is low compared to data reported by Han et al. (71) in Americans from 2009 to 2015 showing that the 12-month prevalence of SI increased from 6.1 to 8.3%. In a 12-month prevalence study in Spanish university students, results showed that around 9.9% had experienced SI (58). In this regard, and even though MEmind is an app that offers participants a space where they can freely express themselves (59), it is possible that in our study some college students were not completely sincere when reporting their responses about SI. Some of the researchers were also professors in their university, and, therefore, some of the students might have been reluctant to share private and sensitive information. In this direction, Schmeelk-Cone et al. (72) highlighted that students are often uncertain about talking to teachers about private problems, including the presence of SI. Specifically, Pisani et al. (73), in their study that included 381 participants who had considered committing SA in the past, found that only about one in four students told this intention to an adult. Future research should count on collaborators apart from their own university staff, in order to reduce reluctance to openly respond to questions about SI. In addition, it should be noted that of the 975 participants approached in this study, only 737 completed the first assessment. Therefore, 24.4% of the students who did not complete the assessment might have experienced higher rates of SI and, for this reason, could have affected the overall rates of SI in this study.

Moreover, we found a lack of curvature in the trajectory analysis for SI, reflecting that it remained relatively stable weekly over the six-month period in our sample, with no significant upward or downward variations. Some previous studies have revealed mixed results, finding fluctuations in SI (74). In the study by Gratch et al. (75), 58% of their participants who reported SI using EMA methodology denied any past-week ideation. However, in our study, non-significant fluctuations in SI were found when SI was moderate or low in frequency. In the same direction, another study found that people with a multiple attempt status more frequently experienced a wider range and greater intensity of their SI over time, compared to those with limited or no experience of SI (76). In addition, our results are in line with the work by Bernanke et al. (21), and it is possible that participants in our sample may be best described as non-stress responsive ideators because they do not present a pattern of SI associated with rapid, changing, and potentially fleeting fluctuations. Furthermore, Kleiman et al. (22) postulated that there may be two superordinate phenotypes (high versus low variability) of SI. In this regard, our results are in line with their findings, and our sample might be better described as non-stress responsive ideators with low variability in SI.

In addition, for the second objective of exploring the associations between risk and protective factors and their relationships with participants’ inclusion in categories of high, moderate, or low suicide ideation, we expected to find that risk factors better represented the group of participants with high levels of SI. However, only one participant scored high on this variable, and, therefore, it was considered an extreme value. Specifically, we found two groups of latent dimensions related to risk and protective factors against SI. One latent dimension of risk factors better represented the group of participants with moderate levels of SI, and a second latent dimension of protective variables better represented the group of patients with lower levels of SI. Specifically, we found that participants with moderate levels of SI tended to show higher levels of thwarted belongingness, perceived burdensomeness, depressive symptoms, negative affect, and emotional suppression. This finding may indicate that participants in the group with stable moderate levels of SI overall showed higher levels of risk factors. In this regard, for thwarted belongingness and perceived burdensomeness, previous literature has shown that these variables are risk factors related to SI in college samples (77, 78). Furthermore, Lockman and Servaty-Seib (79) showed support for these variables as proximal mental states associated with SI in university students, although they highlighted that perceived burdensomeness was the strongest proximal interpersonal risk factor predicting SI. In fact, Díez-Gómez et al. (80) found four distinct groups related to suicide risk in their latent class analysis: “low risk-healthy,” “suicidal act,” “suicidal ideation,” and “high suicide risk” in a sample of 1,506 adolescent students. Results showed that the group of participants with high theoretical suicide risk showed lower scores on positive affect and higher levels of behavioral and emotional problems. Although our sample is composed of college students, our results are similar because the group of participants with moderate levels of SI had higher levels of negative affect and emotional difficulties.

Moreover, past research has shown that depressive symptoms are an important risk factor related to SI in university students [i.e., (81, 82)]. In the case of emotional dysregulation, our results may indicate that the group of college students that tended to show moderate levels of SI used this strategy as a way of escaping negative emotions or internal states. This relationship has been found in previous literature (83–85). Furthermore, DeShong et al. (77) found that neuroticism, a personality trait characterized by the presence of elevated rates of negative affect, was positively associated with thwarted belongingness and perceived burdensomeness in a sample of college students with SI. Likewise, Bowen et al. (86) found that negative affect and emotional instability independently predicted SI.

In addition, we found that participants in the group with lower levels of SI tended to show higher levels of positive affect, cognitive reappraisal, and purpose in life, indicating that participants in the group with stable lower levels of SI showed higher levels of protective factors. In this regard, Li et al. (78) showed that having a reason for living seemed to be a protective factor against SI and suicidal behaviors in college students. Therefore, the ability to reevaluate life as a value that gives meaning to experience may help students to avoid the presence of SI. Thus, these findings may indicate that students with a sense of having a life worth living who tend to reevaluate their negative cognitions and frequently experience positive affect are less likely to experience high levels of SI. Therefore, these factors would protect them from the presence of these dysfunctional thoughts.

We have to highlight some limitations. First, the trajectories could not confirm a significant improvement in goodness of fit due to the small sample size of Spanish college students. Second, our final sample was composed of 28 of the 737 participants who reported at least one moment of SI during the EMA follow-up, and so the sample that reported SI was quite small. Therefore, the generalization of our results is limited, and these results should be replicated in future research with larger samples. Future studies should explore suicide behaviors and their profiles or classes with the aim of differentiating subtypes within the same construct (e.g., subtypes of people with SI/SA) (87).

Moreover, it is possible that this sample underestimated the real prevalence of SI trajectories in college students, given the possible sincerity problems. Furthermore, it would be important to explore our hypotheses with larger samples of university students. Third, although daily assessment may have practical advantages for this population, the students had Smartphone restrictions while they were attending lectures, and so it is possible that they could not immediately respond to some of the questions, which could have influenced later responses. Finally, we assessed suicide ideation through questions created *ad hoc* by the research team. The use of a validated questionnaire on suicide ideation would have been more appropriate.

Despite these limitations, to the best of our knowledge, this is the first study to evaluate fluctuations in SI in Spanish college students using EMA methodology. Prior research in our country with this population assessed SI using traditional paper-and-pencil interviews (88) or online surveys (58, 89). Moreover, the majority of the EMA studies that evaluated SI integrated a fairly short follow-up time (one or two weeks) (45). In this regard, our follow-up of EMA for six months sheds more light on SI trajectories in non-clinical samples. Thus, our results highlight the need for university units dedicated to suicide prevention. In this regard, evidence shows that acknowledging and talking about suicide may in fact reduce SI (90). Therefore, there is a need to monitor mental health in college students and improve evaluation strategies in order to achieve greater transparency and honesty when reporting SI using digital applications.

## Data Availability Statement

The raw data supporting the conclusions of this article will be made available by the authors, without undue reservation.

## Ethics Statement

Our work was approved by the Ethics Committee of the Catholic University of Valencia (code 072), research code “UCV2017-2018-116”. The patients/participants provided their written informed consent to participate in this study.

## Author Contributions

SP, JM, and EB-G: conception and design of the work. JL: data collection. JL, AC, JM, MB, EB-G, and SP: data analysis and interpretation and critical revision of the manuscript. JL, JM, and SP: drafting the manuscript. All authors contributed to the article and approved the submitted version.

## Conflict of Interest

The authors declare that the research was conducted in the absence of any commercial or financial relationships that could be construed as a potential conflict of interest.

## Publisher’s Note

All claims expressed in this article are solely those of the authors and do not necessarily represent those of their affiliated organizations, or those of the publisher, the editors and the reviewers. Any product that may be evaluated in this article, or claim that may be made by its manufacturer, is not guaranteed or endorsed by the publisher.

## Abbreviations

SI: suicidal ideation
SA: suicide attempts
WHO: World Health Organization
EMA: Ecological Momentary Assessment.
